# Multi-strain probiotic supplement attenuates streptozotocin-induced type-2 diabetes by reducing inflammation and β-cell death in rats

**DOI:** 10.1371/journal.pone.0251646

**Published:** 2021-06-24

**Authors:** Pei-Shan Hsieh, Hsieh-Hsun Ho, Shu Ping Tsao, Shih-Hung Hsieh, Wen-Yang Lin, Jui-Fen Chen, Yi-Wei Kuo, Shin-Yu Tsai, Hui-Yu Huang

**Affiliations:** 1 Functional Research Division, Department of Research and Design, Bioflag Biotech Co., Ltd., Tainan, Taiwan; 2 Department of Drug Discovery and Development Industry, College of Pharmacy, Taipei Medical University, Taipei, Taiwan; 3 Product Division, Department of Research and Design Bioflag Biotech Co., Ltd., Tainan, Taiwan; 4 Graduate Institute of Metabolism and Obesity Sciences, Taipei Medical University, Taipei, Taiwan; Auburn University, UNITED STATES

## Abstract

Probiotics are health beneficial bacterial populations colonizing the human gut and skin. Probiotics are believed to be involved in immune system regulation, gut microbiota stabilization, prevention of infectious diseases, and adjustments of host metabolic activities. Probiotics such as *Lactobacillus* and *Bifidobacterium* affect glycemic levels, blood lipids, and protein metabolism. However, the interactions between probiotics and metabolic diseases as well as the underlying mechanisms remain unclear. We used streptozotocin (STZ)-induced diabetic animal models to study the effect of Probioglu^TM^, a multi-strain probiotic supplement including *Lactobaccilus salivarius* subsp. *salicinius* AP-32, *L*. *johnsonii* MH-68, *L*. *reuteri* GL-104, and *Bifidobacterium animalis* subsp. *lactis* CP-9, on the regulation of physiochemical parameters related to type-2 diabetes. Experimental rats were randomly assigned into five groups, control group, streptozotocin (STZ)-treated rats (STZ group), STZ + 1× Probioglu^TM^ group, STZ + 5× Probioglu^TM^ group, and STZ + 10× Probioglu^TM^ group, and physiological data were measured at weeks 0, 2, 4, 6, and 8. Our results indicate that supplementation with Probioglu^TM^ significantly improved glucose tolerance, glycemic levels, insulin levels, and insulin resistance (HOMA-IR). Furthermore, we observed reduction in urea and blood lipid levels, including low-density lipoprotein (LDL), triglycerides (TG), and total cholesterol (TC). Probioglu^TM^ administration increased the β-cell mass in STZ-induced diabetic animal models, whereas it reduced the levels of proinflammatory cytokines TNF-α, IL-6, and IL-1β. In addition, the enhancement of oxidative stress biomarkers and superoxide dismutase (SOD) activities was associated with a decrease in malondialdehyde (MDA) levels. We conclude that Probioglu^TM^ attenuates STZ-induced type-2 diabetes by protecting β-cells, stabilizing glycemic levels, and reducing inflammation. Among all probiotic treating groups, the 10×Probioglu^TM^ treatment revealed the best results. However, these experimental results still need to be validated by different animal models of type-2 diabetes and human clinical trials in the future.

## Introduction

Metabolic syndromes, including obesity, coronary heart diseases, stroke, hyperuricemia, chronic kidney diseases, and diabetes, tend to occur at younger ages and recently have become one of the biggest global health problems, especially in developed and developing countries [1]. Metabolic disorder-related diseases can directly or indirectly interact with other conditions (i.e., obesity dramatically increases the risk of type-2 diabetes [2], or diabetes associated with cardiovascular diseases) [3], thus making their control more difficult. Diabetes is a severe metabolic syndrome closely related to various complications such as chronic kidney disease, blindness [4], and Alzheimer’s disease [5]. Numerous drugs are available to control glycemic levels in diabetes patients; however, they usually have various side effects, such as gastrointestinal distress, nausea, and diarrhea, that lower quality of life.

Probiotics are symbiotic microorganisms that reside naturally in the human skin, gut, respiratory tract, genital tract, and mucosal tissues [6]. Their populations are markedly affected by the daily lifestyle of the host, and any consequent changes can cause sub-health conditions such as atopic dermatitis, obesity, asthma, allergic diseases, inflammatory bowel disease, and oral ulcers. The regular human dosage for daily probiotic consumption is around 1* 10^10^ [7].

Chronic low-grade inflammation mediated by M1-type adipose tissue macrophages (CD8 T and Th1 cells) in long-term overweight or obese individuals can result in insulin resistance and type-2 diabetes [8]. Diabetes patients are estimated to account for approximately 8% of the global population [9], of which 95% has type-2 diabetes. Previous studies have shown that the gut microbiota is involved in energy metabolism and associated with metabolic disorders such as obesity and type-2 diabetes; however, this interrelationship is affected by genetic and environmental factors.

We have previously shown that certain probiotic strains reduce blood glucose levels in mice [10]. Therefore, in this study, we selected probiotic strains *Lactobaccilus salivarius* subsp. salicinius AP-32, *L*. *johnsonii* MH-68, *L*. *reuteri* GL-104, and *Bifidobacterium animalis* subsp. lactis CP-9 as major components of a probiotic product and investigated its effects on a type-2 diabetic rat model obtained with STZ treatment and a high-fat diet [11, 12]. Glycemic index, insulin levels, beta-cell mass, blood lipid levels, and anti-oxidant activity were determined.

## Materials and methods

### Animals

All experiments and protocols complied with the Laboratory Animal Care and Use Guidelines published by the Taiwan government. The protocols were approved by the Shih Chien University Animal Ethics Committee (Permit no. 10803). Male Sprague Dawley (SD) rats were purchased from BioLasco (Taiwan) and housed at the Laboratory Animal Center, Shih Chien University, Taipei, Taiwan, under controlled conditions (12-h light/12-h dark cycle, 22°C ± 2°C, and 62% ± 5% humidity). The animals were provided with sterilized water and food throughout the experimental period (weeks 1–8).

In this experiment, 50 six-week-old male SD rats were purchased from Lesco, originally 10 in each group. At 0 weeks, 2 rats in each group with poor mobility were exclude (e.g. rats with no responding of grasp), and finally 8 male SD rats were in each group. The standard for animal humane suspension of the experiment is to observe the physiological state of the animal every day during the experiment to see if there is any abnormal behavior of weakness, motionlessness, stopping water or eating. If there is any abnormal behavior, the experiment shall be terminated humanely in advance. The groups are: normal control group (Normal Control, N) and diabetic hyperglycemia group as negative control group (Diabetes Mellitus, DM), probiotics 1X group (DM1X), probiotics 5X group (DM5X), probiotics Bacteria 10X group (DM10X). Groups with induced diabetic hyperglycemia include DM, DM1X, DM5X, DM10X.

### Streptozotocin-induced diabetes animal model

SD rats were administered with nicotinamide (NA, 30–60 mg kg-1), 15–30 min before treatment with 10–20 mg kg-1 streptozotocin (STZ) to deactivate β-cells in the pancreas. NA and STZ were injected intraperitoneally every 2 d for 8 weeks. To further elicit diabetes, STZ-treated rats were fed with a high-fat diet (Research Diets, D12492) [13]. General anesthesia is required for most methods of blood collection in rats, to prevent restraint. Rats were anesthetized with an intraperitoneal injection of Zoletil/Xylazine (20~40mg/kg Z+5-10mg/kg X). Fasting blood glucose and fasting blood insulin levels were monitored every 2 weeks. The blood samples were collected from the tail of rats.

### Probiotic treatments

The multi-strain probiotic “ProbiogluTM” (*Lactobaccilus salivarius* subsp. *salicinius* AP-32, 2.5 × 10^9^ CFU capsule^-1^; *L*. *johnsonii* MH-68, 2.5 × 10^9^ CFU capsule^-1^; *L*. *reuteri* GL-104, 2.5 × 10^9^ CFU capsule^-1^; and *Bifidobacterium animalis* subsp. *lactis* CP-9, 2.5 × 10^9^ CFU capsule^-1^) was provided by Bio-flag Biotech (Taiwan). STZ-treated rats were orally administered with 1,000 μL gavages containing different quantities of Probioglu^TM^ (DM1X: STZ + 1× Probioglu^TM^, 5.17 × 10^9^ CFU kg^-1^ d^-1^; DM5X: STZ + 5× Probioglu^TM^, 2.58 × 10^10^ CFU kg^-1^ d^-1^; DM10X: STZ + 10× Probioglu^TM^, 5.17 × 10^10^ CFU kg^-1^ d^-1^) once a day for 8 weeks. Untreated (N: control group) and STZ-treated (DM: STZ group) rats without probiotic administration were used as a reference. 8 rats were randomly assigned to each group. The animals were sacrificed to collect serum and pancreas samples for analysis.

### Evaluation of body weight, food consumption, and water intake

Body weights were measured twice a day at 0, 2, 4, 6, and 8 weeks. For evaluation of the daily food consumption, 35 g of fodder was provided, and the remaining was weighed after 24 h. Data on the daily water intake were collected using a measuring bottle. Body weight, food consumption, and water intake observed in STZ and STZ + Probioglu^TM^ groups were compared with the control group.

### Fasting blood glucose (FBG) and insulin investigation

FBG and fasting blood insulin levels were monitored at 0, 2, 4, 6, and 8 weeks. Blood samples were collected from the tail of the rats. Glycemic levels were measured using the Optium Xceed meter (Abbott Diabetes Care, USA) with a blood glucose test strip. The blood insulin concentrations were detected using the Rat Insulin ELISA Kit (Mercodia, Sweden). Insulin concentrations were further converted into enzyme activities (1 μg L^-1^ = 24 μU ml^-1^) [14, 15]. The Homeostasis Model Assessment-Insulin Resistance (HOMA-IR) was calculated as follows:

HOMA-IR blood glucose (mmol L^-1^) × insulin (mU L^-1^)/22.5 [16].

### Oral glucose tolerance test (OGTT)

For OGTT, all groups were orally administered with 1 ml glucose (1 g kg^-1^) at weeks 4 and 8, and their blood glucose and insulin levels were analyzed at 30 min, 60 min, 90 min, 120 min, and 180 min after administration. Blood glucose was detected using a rat glucose assay kit (Randox, UK), whereas insulin concentrations were analyzed using the Rat Insulin ELISA Kit (Mercodia, Sweden). The total glucose area under the curve (AUC) was grouped and calculated at the time periods of 0–30 min, 30–60 min, 60–90 min, 90–120 min, and 120–180 min as described previously [17].

### Determination of β-cell mass

The β-cell mass in the pancreas was detected as described previously [18]. Briefly, immunohistochemical staining of pancreatic sections was performed to determine the area of pancreatic islets. Each pancreas sample was sliced into 50 sections (12 μm thick for each section). The areas of pancreatic islets and total pancreas were analyzed by microscopy and quantified by ImageJ (https://imagej.net/ImageJ). To further calculate the total number of pancreatic cells and that of β-cells, hematoxylin and eosin stains were applied. The cell numbers were counted under 200× magnification of an optical microscope. The β-cell mass was calculated as follows:

β-cell mass = weight of the pancreas × (number of β-cells/total number of pancreatic cells)/area of the pancreatic section slide.

The experiments were performed by two independent researchers to prevent bias.

### Serum biochemistry

Blood samples were immediately centrifuged after collection and stored at −80°C until evaluation. Serum triglyceride (TG), total cholesterol (TC), low-density lipoprotein (LDL), high-density lipoprotein (HDL), free fatty acids (FFA), and urea were evaluated using commercially available kits (UR-107, Randox, UK) and analyzed via the Beckman Coulter Automated Chemistry Analyzer (AU680, Non‐Sterile, Beckman Coulter, USA). Proinflammatory cytokines, including TNF-α, IL-6, and IL-1β, were evaluated using commercial ELISA kits (BioLegend, USA; Peprotech, USA). The concentrations of cytokines were measured using the Sunrise ELISA Reader (Tecan, Switzerland).

### Evaluation of oxidative stress bioindicators

Bioindicators reflecting oxidative stress, including superoxide dismutase (SOD; Cayman Chemical, Item No. 706002), glutathione peroxidase (GSH; Cayman Chemical Item No. 703102), and malondialdehyde (MDA; Cayman Chemical, Item No. 10009055) were analyzed using commercial assay kits (Cayman Chemical, Michigan, USA). SOD, GSH, and MDA were quantified by measuring absorbance at 450 nm, 340 nm, and 520 nm, respectively.

### Statistical analysis

Statistical analysis was performed using Microsoft Excel and Prism 8 (GraphPad, USA). Data are presented as means ± standard deviation (SD) obtained from two or three independent experiments and collected from eight animals. Differences were identified using one-way analysis of variance in conjunction with the Duncan’s new multiple range test (MRT) and considered significant at *p* < 0.05.

## Results

### Probioglu^TM^ induced dose-dependent restoration of food consumption and water intake in STZ-treated rats

**Fig 1:**
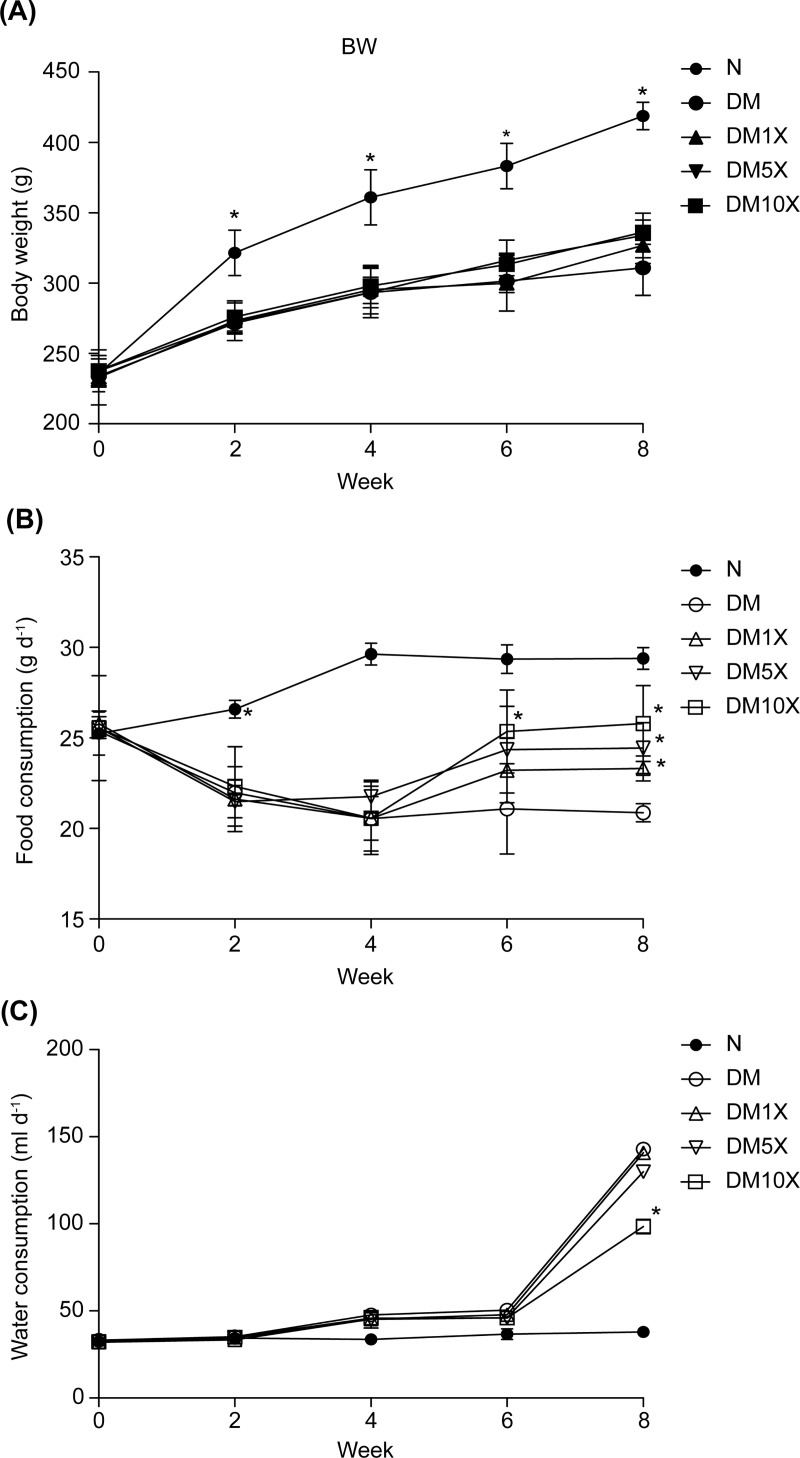
(A) Body weight, (B) food consumption, and (C) water intake of untreated rats (control group), streptozotocin (STZ)-treated rats (STZ group), and STZ + Probioglu^TM^-treated rats (STZ + 1× Probioglu^TM^ group, 5.17 × 10^9^ CFU kg-1 d-1; STZ + 5× Probioglu^TM^ group, 2.58 × 10^10^ CFU kg-1 d-1; STZ + 10× Probioglu^TM^ group, 5.17 × 10^10^ CFU kg-1 d-1) at weeks 0, 2, 4, 6, and 8 of the experimental period. Each group consisted of 8 rats in this study. * indicates significant differences compared with the control at p < 0.05.

In this study, type-2 diabetic rats were induced by low-dose STZ (10–20 mg kg^-1^) and a high-fat diet. Body weight, food consumption, and water intake were monitored twice daily at weeks 0, 2, 4, 6, and 8 in all groups (Fig 1). The body weight and food consumption of STZ and STZ + Probioglu^TM^ groups were significantly lower than those of the control group (Fig 1A and 1B); however, Probioglu^TM^ treatment led to a dose-dependent restoration of appetite compared to the DM group at week 8 (DM group with STZ treatment only: 20.87 ± 0.5 g; DM10X: 25.79 ± 2.1 g: p < 0.05 *; Fig 1B). Water intake was significantly higher in the STZ and STZ + Probioglu^TM^ groups compared with the control group at week 8 (Control: 39.91 ± 1.7 mL; DM10X: 98.38± 4.2 mL; p<0.05 *; Fig 1C). The restoration of water intake was only observed in the STZ + 10× Probioglu^TM^ group (Fig 1C).

### Probioglu^TM^ reduced glycemic levels and partially restored glucose tolerance in STZ-treated rats

Glycemic levels were monitored throughout the experimental period in all groups (Table 1). Compared with the control group, FBG levels increased in all STZ groups at weeks 2–8; at week 8, glycemic were 3-fold higher in the STZ group (DM: 295.6 ± 9.8 mg/dL) compared with the control group (N: 96.7 ± 9.8 mg/dL). However, the rate of increase was significantly slower in the STZ + 10× Probioglu^TM^ group (DM10X at week 8: 220.5 ± 9.3 mg/dL; p < 0.05 *) than in the STZ group.

**Table 1:** Glycemic levels of rats in each group at weeks 0, 2, 4, 6, and 8.

Week	Control (mg dL^-1^)	STZ (mg dL^-1^)	STZ + 1× Probioglu^TM^ (mg dL^-1^)	STZ + 5 × Probioglu^TM^ (mg dL^-1^)	STZ + 10× Probioglu^TM^ (mg dL^-1^)
**0**	87.3 ± 2.5	87.6 ± 2.2	88.5 ± 1.2	87.4 ± 2.5	87.7 ± 2.4
**2**	90.1 ± 1.2	141.7 ± 5.7#	128.3 ± 9.9#	124.9 ± 5.1#	120.6 ± 7.0#*
**4**	92.6 ± 1.4	257.0 ± 23.4#	234.5 ± 16.2#*	212.7 ± 38.1#*	209.2 ± 37.8#*
**6**	96.2 ± 1.7	280.9 ± 19.1#	248.2 ± 17.5#*	229.1 ± 24.9#*	219.4 ± 19.5#*
**8**	96.7 ± 3.4	295.6 ± 9.8#	255.9 ± 12.9#*	240.2 ± 17.4#*	220.5 ± 9.3#*

Untreated (control group), streptozotocin (STZ)-treated rats (STZ group), and STZ + ProbiogluTM-treated rats (STZ + 1× ProbiogluTM group, 5.17 × 10^9^ CFU kg^-1^ d^-1^; STZ + 5× Probioglu^TM^ group, 2.58 × 10^10^ CFU kg^-1^ d^-1^; STZ + 10× Probioglu^TM^ group, 5.17 × 10^10^ CFU kg^-1^ d^-1^). Each group contained 8 rats.

* indicates a significant difference compared with the STZ group at *p* < 0.05

# represents a significant difference compared to the control group at *p* < 0.05.

**Fig 2:**
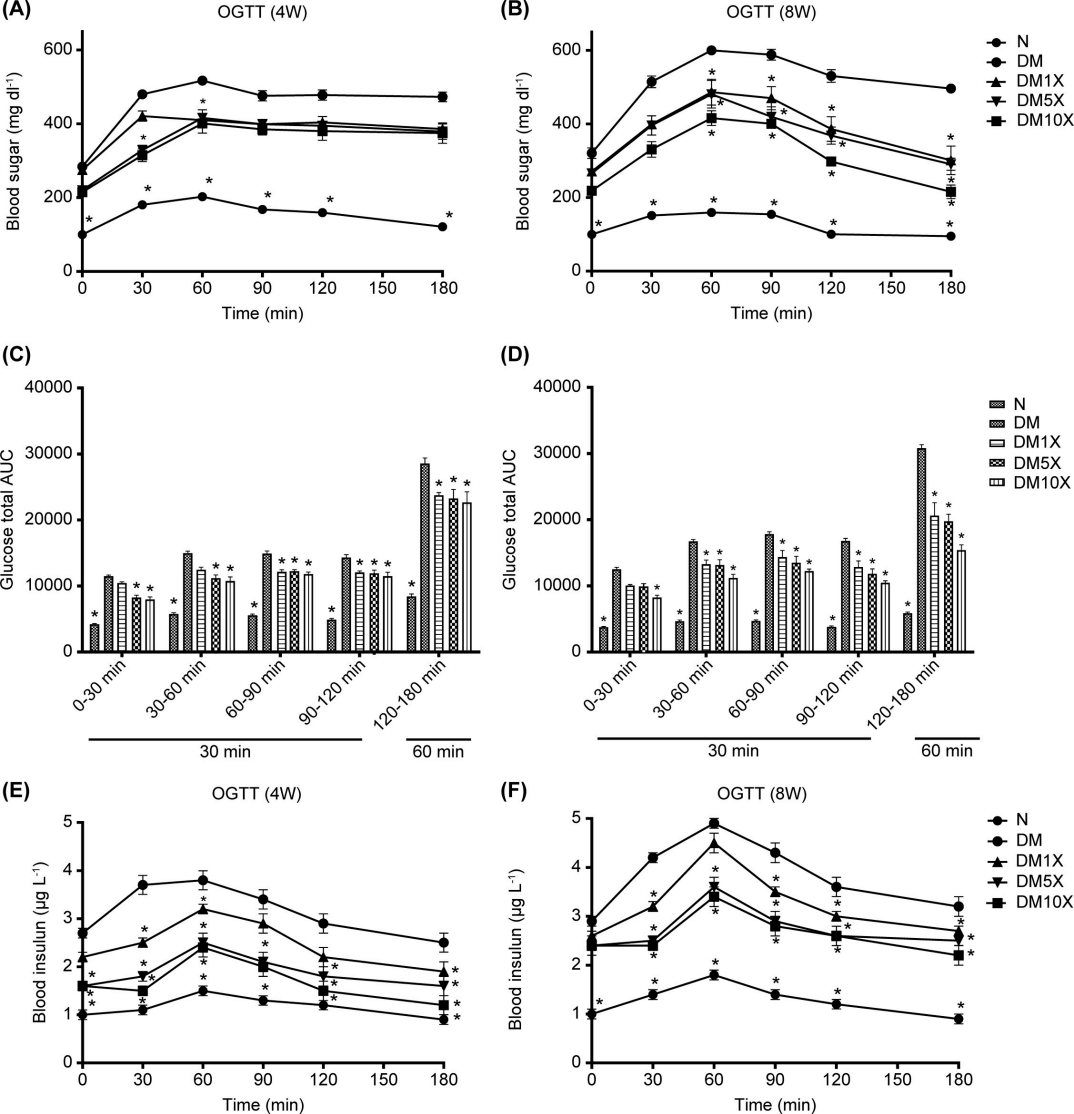
(A) Blood sugar at week 4 and (B) week 8, (C) glucose total area under the curve (AUC) at week 4 and (D) week 8, (E) blood insulin at week 4, and (F) week 8, and of untreated rats (control group), streptozotocin (STZ)-treated rats (STZ group), and STZ + Probioglu^TM^-treated rats (STZ + 1× Probioglu^TM^ group, 5.17 × 10^9^ CFU kg^-1^ d^-1^; STZ + 5× Probioglu^TM^ group, 2.58 × 10^10^ CFU kg^-1^ d^-1^; STZ + 10× Probioglu^TM^ group, 5.17 × 10^10^ CFU kg^-1^ d^-1^). Each group consisted of 8 rats in this study. Blood sugar and insulin levels were analyzed at 0, 30, 60, 90, 120, 180 min after the administration of high-dose glucose (1 g kg^-1^).* indicates significant differences compared with the control at *p* < 0.05.

OGTT were evaluated at weeks 4 and 8 in all groups (Fig 2). In the control group, blood sugar levels showed small increases at 60 min after the administration of high-dose glucose and decreased at 120 min at weeks 4 and 8. All STZ groups showed relatively high levels of blood sugar before glucose uptake, whereas the level of blood sugar failed to decrease at 60 min after the administration of high-dose glucose at week 4 (Fig 2A). However, OGTT data at week 8 showed that blood glucose levels in the Probioglu^TM^ uptaking group at 180 min returned to the levels at 0 min (DM10X at 0 min: 218.6 ± 5.1 mg/dL; DM10X at 180 min: 215.6 ± 18.5 mg/dL; Fig 2B). Further analysis of the total AUC during OGTT is shown in Fig 2C and 2D.

### Probioglu^TM^ reduces blood insulin levels and insulin resistance (HOMA-IR) in STZ-treated rats

Insulin levels were monitored throughout the experimental period in all groups (Table 2). Compared with the control group, fasting blood insulin levels increased in all STZ groups at weeks 2–8. At week 8, insulin levels were 3-fold higher in the STZ group than in the control group (STZ group: 3.38 ± 0.15 μg L^-1^; control group: 1.08 ± 0.03 μg L^-1^; p < 0.05*). However, the rate of increase was significantly lower in the STZ + 10× Probioglu^TM^ group (2.18 ± 0.12 μg L^-1^, p < 0.05 *).

**Table 2:** Insulin levels of rats in each group at weeks 0, 2, 4, 6, and 8.

Week	Control (μg l^-1^)	STZ (μg l^-1^)	STZ + 1× Probioglu^TM^ (μg l^-1^)	STZ + 5× Probioglu^TM^ (μg l^-1^)	STZ + 10× Probioglu^TM^ (μg l^-1^)
**0**	0.90 ± 0.05	0.90 ± 0.06	0.93 ± 0.04	0.91 ± 0.05	0.90 ± 0.06
**2**	0.92 ± 0.03	1.93 ± 0.09#	1.89 ± 0.08 #	1.69 ± 0.13#*	1.50 ± 0.2 #*
**4**	0.95 ± 0.03	2.35 ± 0.28#	2.13 ± 0.10#*	1.76 ± 0.14#*	1.56 ± 0.19#*
**6**	1.01 ± 0.04	2.42 ± 0.29#	2.20 ± 0.07#*	1.97 ± 0.16#*	1.63 ± 0.16#*
**8**	1.08 ± 0.03	3.38 ± 0.15#	2.61 ± 0.10#*	2.35 ± 0.06#*	2.18 ± 0.12#*

Untreated rats (control group), streptozotocin (STZ)-treated rats (STZ group), and STZ + ProbiogluTM-treated rats (STZ + 1× Probioglu^TM^ group, 5.17 × 10^9^ CFU kg^-1^ d^-1^; STZ + 5× Probioglu^TM^ group, 2.58 × 10^10^ CFU kg^-1^ d^-1^; STZ + 10× Probioglu^TM^ group, 5.17 × 10^10^ CFU kg^-1^ d^-1^). Each group consisted of 8 rats in this study.

* indicates a significant difference compared with the STZ group at *p* < 0.05

# represents a significant difference compared to the control group at *p* < 0.05.

Blood insulin levels were monitored during OGTT at weeks 4 and 8 in all groups (Fig 2). In the control group, insulin levels showed a small increase at 60 min after the administration of a high-dose of glucose and decreased at 120 min at weeks 4 and 8. Relatively high levels of insulin were consistently observed in all STZ groups before and after glucose administration (Fig 2E and 2F). In contrast, 4 weeks after supplying Probioglu^TM^, insulin levels at 60 min were partially maintained, and the rate of increase was lower than in the STZ group (DM10X: 2.4 ± 0.2 μg L^-1^; STZ: 3.8 ± 0.2 μg L^-1^; p < 0.05 *; Fig 2E). Eight weeks after Probioglu^TM^ treatment, the animals showed a similar tendency (DM10X: 3.4 ± 0.2 μg L^-1^; STZ: 4.9 ± 0.1 μg L^-1^; p < 0.05*; Fig 2F). In addition, the insulin resistance indicator, HOMA-IR, was 10-fold higher in the STZ group than in the control group (control: 6.15 ± 0.3; STZ: 59.17 ± 3.1; p < 0.05#), whereas Probioglu^TM^ supplementation significantly lowered insulin resistance at least 33% (STZ: 59.17 ± 3.1; 1× Probioglu^TM^: 39.52 ± 3.1; p < 0.05, Table 3).

**Table 3:** Serum chemical analysis of rats in each group at the end of the experimental period.

Parameter	Control	STZ	STZ + 1× Probioglu^TM^	STZ + 5 × Probioglu^TM^	STZ + 10× Probioglu^TM^
**TG (mg dl**^**-1**^**)**	52.6 ± 12.4	325.8 ± 18.2#	315 ± 16.5#	203.5 ± 12.7#*	180.2 ± 12.6#*
**TC (mg dl**^**-1**^**)**	53.4 ± 10.2	115.2 ± 62.6#	69.8 ± 22.4*	66.2 ± 18.6*	62.9 ± 17.4*
**LDL (mg dl**^**-1**^**)**	38.5 ± 11.6	72.5 ± 23.1#	63.2 ± 18.7#	49.0 ± 15.4*	40.5 ± 10.4*
**HDL (mg dl**^**-1**^**)**	1.76 ± 0.23	1.62 ± 0.30	1.67 ± 0.28	1.68 ± 0.27	1.64 ± 0.36
**FFA (μg ml**^**-1**^**)**	43.3 ± 3.8	49.5 ± 7.4	42.9 ± 9.5	44.5 ± 4.3	42.6 ± 6.2
**Urea (mg dl**^**-1**^**)**	11.3 ± 5.8	44.1 ± 11.6#	38.3 ± 8.2#	26.1 ± 11.7#	21.8 ± 9.3#
**HOMA-IR**	6.15 ± 0.3	59.17 ± 3.1#	39.52 ± 3.1#*	33.45 ± 2.8#*	28.39 ± 2.1#*

Untreated rats (control group), streptozotocin (STZ)-treated rats (STZ group), and STZ + Probioglu^TM^-treated rats (STZ + 1× Probioglu^TM^ group, 5.17 × 10^9^ CFU kg^-1^ d^-1^; STZ + 5× Probioglu^TM^ group, 2.58 × 10^10^ CFU kg^-1^ d^-1^; STZ + 10× Probioglu^TM^ group, 5.17 × 10^10^ CFU kg^-1^ d^-1^). Each group consisted of 8 rats in this study.

TG, triglyceride; TC, total cholesterol; LDL, low-density lipoprotein; HDL, high-density lipoprotein.

FFA, free fatty acids; HOMA-IR, homeostasis model assessment-insulin resistance index.

* indicates a significant difference compared with the STZ group at *p* < 0.05

# represents a significant difference compared to the control group at *p* < 0.05.

### Probioglu^TM^ attenuated β-cell death and increased β-cell mass in STZ-treated rats

**Fig 3:**
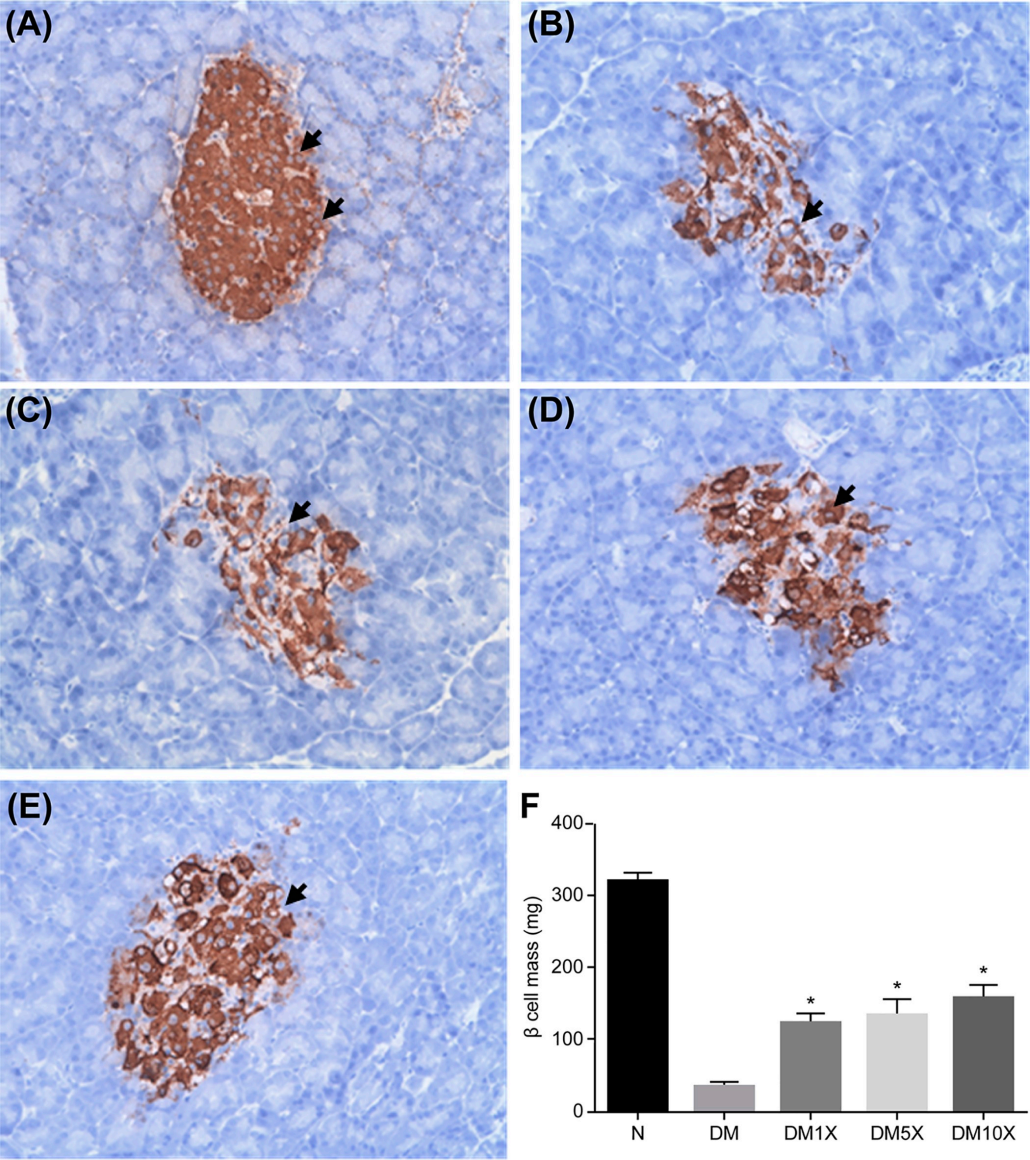
Microscopic image of the pancreas of (A) untreated rats (N, control group), (B) streptozotocin (STZ)-treated rats (DM, STZ group), and (C–E) STZ + Probioglu^TM^-treated rats (DM1X: STZ + 1× Probioglu^TM^ group, 5.17 × 10^9^ CFU kg^-1^ d^-1^; DM5X: STZ + 5× Probioglu^TM^ group, 2.58 × 10^10^ CFU kg^-1^ d^-1^; DM10X: STZ + 10× Probioglu^TM^ group, 5.17 × 10^10^ CFU kg^-1^ d^-1^) at the end of the experimental period. (F) Mean β-cell mass in each group at the end of the experimental period. Each group consisted of 8 rats in this study. * indicates significant differences compared with the control: *p* < 0.05.

Beta-cell death and beta-cell mass was evaluated at the end of the experimental period in all groups (Fig 3). At week 8, pancreatic islets of STZ groups were smaller than those of the control group and also impaired (control: 320.9 ± 10.4 mg; STZ: 35.3 ± 5.4 mg; p < 0.05*; Fig 3A, 3B and 3F); however, supplementation with Probioglu^TM^ attenuated STZ-induced β-cell death in a dose-dependent manner, since some pancreatic regions remained intact (DM1X: 124.8 ± 11.2 mg; DM5X: 135.6 ± 20.3 mg; DM10X: 158 ± 17.7 mg; Fig 3C–3F) and the decrease in β-cell mass was markedly lower (p < 0.05*; Fig 3F).

### Probioglu^TM^ improved serum biochemistry indices in STZ-treated rats

Serum biochemistry and bioindicators of oxidative stress were evaluated at the end of the experimental period in all groups (Tables 3 and 4). Compared with the control group, triglyceride (TG) was 6-fold higher in the STZ group but less than 4-fold higher in the STZ + 5× Probioglu^TM^ and STZ + 10× Probioglu^TM^ groups (control: 52.6 ± 12.4 mg dL^-1^; STZ: 325.8 ± 18.2 mg dL^-1^; STZ + 1× Probioglu^TM^: 315 ± 16.5 mg dL^-1^; STZ + 5× Probioglu^TM^: 203.5 ± 12.7 mg dL^-1^; STZ + 10× Probioglu^TM^: 180.2 ± 12.6 mg dL^-1^; Table 3). Compared with the control group, total cholesterol (TC) was significantly higher in the STZ group, and feeding STZ + Probioglu^TM^ groups showed significantly reduced levels of TC (control: 53.4 ± 10.2 mg dL^-1^; STZ: 115.2 ± 62.6 mg dL^-1^; STZ + 1× Probioglu^TM^: 69.8 ± 22.4 mg dL^-1^; STZ + 5× Probioglu^TM^: 66.2 ± 18.6 mg dL^-1^; STZ + 10× Probioglu^TM^: 62.9 ± 17.4 mg dL^-1^; Table 3). LDL was significantly higher in the STZ group compared with the control, whereas Probioglu^TM^ supplementation improved the levels in a dose-dependent manner (control: 38.5 ± 11.6 mg dL^-1^; STZ: 72.5 ± 23.1 mg dL^-1^; STZ + 1× Probioglu^TM^: 63.2 ± 18.7 mg dL^-1^; STZ + 5× Probioglu^TM^: 49.0 ± 15.4 mg dL^-1^; STZ + 10× Probioglu^TM^: 40.5 ± 10.4 mg dL^-1^; Table 3). Both HDL and FFA showed no significant differences in any group (Table 3). Urea levels were significantly higher in all STZ groups compared with the control group, and supplementation with Probioglu^TM^ showed only partial improvements in a dose-dependent manner (control: 11.3 ± 5.8 mg dL^-1^; STZ: 44.1 ± 11.6 mg dL^-1^; STZ + 1× Probioglu^TM^: 38.3 ± 8.2 mg dL^-1^; STZ + 5× Probioglu^TM^: 26.1 ± 11.7 mg dL^-1^; STZ + 10× Probioglu^TM^: 21.8 ± 9.3 mg dL^-1^; Table 3).

**Table 4:** Proinflammatory cytokines and oxidative stress bio-indicators of rats in each group at the end of the experimental period.

Parameter	Control	STZ	STZ + 1× Probioglu^TM^	STZ + 5 × Probioglu^TM^	STZ + 10× Probioglu^TM^
**TNF-α (pg ml**^**-1**^**)**	3.4 ± 0.18	4.22 ± 0.51#	3.87 ± 0.61	3.83 ± 0.29	2.83 ± 0.99*
**IL-1β (pg ml**^**-1**^**)**	212.3 ± 84.1	300.2 ± 133.0#	208.7 ± 64.2*	201.6 ± 26.5*	188.6 ± 24.8*
**IL-6 (pg ml**^**-1**^**)**	166.0 ± 32.4	252.5 ± 26.7#	213.4 ± 61.3#*	198.8 ± 51.4*	187.5 ± 35.9*
**SOD activity (U ml**^**-1**^**)**	3.32 ± 0.33	2.26 ± 1.29#	2.64 ± 0.99	3.15 ± 0.69	3.41 ± 0.64*
**MDA (μM)**	1.82 ± 0.06	2.62 ± 0.18#	2.56 ± 0.15#	2.43 ± 0.37#	2.20 ± 0.32*
**GSH (μM)**	7.08 ± 0.75	6.43 ± 2.39	7.73 ± 1.55	7.88 ± 3.29	8.18 ± 2.20

Untreated rats (control group), streptozotocin (STZ)-treated rats (STZ group), and STZ + ProbiogluTM-treated rats (STZ + 1× ProbiogluTM group, 5.17 × 10^9^ CFU kg^-1^ d^-1^; STZ + 5× ProbiogluTM group, 2.58 × 10^10^ CFU kg-1 d-1; STZ + 10× ProbiogluTM group, 5.17 × 10^10^ CFU kg-1 d-1). Each group consisted of 8 rats in this study.

SOD, superoxide dismutase; MDA, malondialdehyde; GSH, glutathione.

* indicates a significant difference compared with the STZ group at *p* < 0.05

# represents a significant difference compared to the control group at *p* < 0.05.

### Probioglu^TM^ attenuated serum proinflammatory cytokines

Proinflammatory cytokines and oxidative stress bioindicators were evaluated at the end of the experimental period in all groups (Table 4). TNF-α was significantly higher in the STZ group than in the control, whereas Probioglu^TM^ supplementation improved the levels in a dose-dependent manner (control: 3.4 ± 0.18 pg mL^-1^; STZ: 4.22 ± 0.51 pg mL^-1^; STZ + 1× Probioglu^TM^: 3.87 ± 0.61 pg mL^-1^; STZ + 5× Probioglu^TM^: 3.83 ± 0.29 pg mL^-1^; STZ + 10× Probioglu^TM^: 2.83 ± 0.99 pg mL^-1^; Table 4). Compared with the control, IL-1β was significantly increased in the STZ group, and uptaking Probioglu^TM^ significantly reduced levels of IL-1β (control: 212.3 ± 84.1 pg mL^-1^; STZ: 300.2 ± 133 pg mL^-1^; STZ + 1× Probioglu^TM^: 208.7 ± 64.2 pg mL^-1^; STZ + 5× Probioglu^TM^: 201.6 ± 26.5 pg mL^-1^; STZ + 10× Probioglu^TM^: 188.6 ± 24.8 pg mL^-1^; Table 4). IL-6 was significantly higher by 50% in the STZ group than that in the control group, whereas Probioglu^TM^ supplementation alleviated the increase in a dose-dependent manner (control: 166.0 ± 32.4 pg mL^-1^; STZ: 252.5 ± 26.7 pg mL^-1^; STZ + 1× Probioglu^TM^: 213.4 ± 61.3 pg mL^-1^; STZ + 5× Probioglu^TM^: 198.8 ± 51.4 pg mL^-1^; STZ + 10× Probioglu^TM^: 187.5 ± 35.9 pg mL^-1^; Table 4).

### Probioglu^TM^ anti-oxidative effects

Compared with the control, superoxide dismutase (SOD) levels were lower in the STZ group, whereas no differences were observed in the STZ + 10× Probioglu^TM^ group (control: 3.32 ± 0.33 U mL^-1^; STZ: 2.26 ± 1.29 U mL^-1^; STZ + 10× Probioglu^TM^: 3.41 ± 0.64 U mL^-1^; Table 4). Malondialdehyde (MDA) levels increased in the STZ group compared with the control. In contrast, the STZ + 10× Probioglu^TM^ significantly alleviated the oxidative stress levels of MDA (control: 1.82 ± 0.06 μM; STZ: 2.62 ± 0.18 μM; STZ + 10× Probioglu^TM^: 2.20 ± 0.32 μM; Table 4). No significant differences were observed in glutathione (GSH) levels in any of the groups (Table 4).

## Discussion

Two main types of diabetes, type 1 and type 2, are caused by relative or absolute insulin insufficiency. Autoimmune attack of insulin-generating pancreatic β-cells leads to the former type, whereas impaired compensation of β-cells leads to the latter type [19]. Two major animal models of type-2 diabetes, the obese and the non-obese, can mimic insulin resistance and β-cell failure [20, 21]. Natural mutations, genetic manipulation, and high-fat feeding are used to develop the obese model. Examples of type-2 diabetes animal models owing to defective leptin receptor-induced obesity are Lepob/ob mouse [22], Leprdb/db mouse [23], and Zucker diabetic fatty rat [24], However, more accurate symptoms and complications of human type-2 diabetes are rendered from polygenic models such as KK mice that present severe hyperinsulinemia, insulin resistance, and diabetic nephropathy [25]; the Otsuka Long-Evans Tokushima Fat rat (OLETF) that demonstrates mild obesity, late-onset hyperglycemia, fibrotic islets, and renal complications [26]; and the New Zealand Obese (NZO) mice that exhibit hyperphagia, obesity, leptin resistance, hyperinsulinemia, elevated blood glucose levels, and hyperplastic islets [27].

Type-2 diabetes animal models are also developed when a high-fat diet (58% of the energy derived from fat compared with 11% of the energy derived from fat in a standard diet) is administered for several weeks, thereby leading to significant weight gain associated with insulin resistance and impaired glucose tolerance [28]. In the present study, a high-fat diet accompanied with low-dose STZ was used to develop type-2 diabetic rats with progressive disease symptoms similar to those in humans, including hyperinsulinemic dysglycemia, hepatic fibrosis, pancreatic β-cell dysfunction, late-stage hyperglycemia, dyslipidemia, decreased myocardial glucose utilization, and renal dysfunction [29].

Streptozotocin (STZ) is commonly used for targeting insulin producing beta cells in pancreas to induce hyperglycemia and mimic T2DM disease conditions. Conventionally, a dosage of 50mg/kg is used [30]. Since STZ is toxic to beta cells, this dosage has been shown to result in necrosis and undesirable high beta cell mortality [30]. In order to mitigate toxicity severity and cell mortality (but the beta cells weren’t completely damaged), we implemented 10-20mg/kg dosage in every two days for 8 days. This animal model was optimized according to previous study, in which multiple low doses of STZ and high-fat diet (HFD) was followed to induce T2DM [30, 31]. Therefore, this optimized method was primarily designed to induce slow and stable damage of beta cells to generate T2DM model rats(S2 Table in S1 Text).

In the present study, we administered different amounts of Probioglu^TM^ to STZ-treated rats to investigate its effects on the regulation of physicochemical parameters related to type-2 diabetes. T2DM induced rats often exhibit the symptoms of polydipsia, polyuria and polyphagia [32]. At present results (Fig 1) clearly shows that the STZ-induced diabetic rats consumed more food, water, which is in line with the previous studies. To explain this phenomenon, hyperglycemia results in escalation of filtered glucose load, causing expulsion of more glucose in urine [33, 34]. Hence, hyperglycemia induces production of high glucose containing urine. The loss of body fluid and high blood glucose lead to tissue dehydration. Therefore, the STZ administered rats are likely to generate a higher volume of urine (polyuria) and urinate more often. The deprivation of water in the tissues might make the rats very thirsty (polydipsia) and trigger signals to drink more water for preventing dehydration. It might be the reason of the rise in water consumption over the last two weeks (Fig 1).

We found that Probioglu^TM^ improved the glycemic index, glucose tolerance (Table 1, Fig 2A–2D), and insulin levels (Table 2 and Fig 2E and 2F), also reduced insulin resistance in HOMA-IR (Table 4). A previous study demonstrated that probiotic treatment with *L*. *salivarius subsp*. *salicinius* AP-32, *L*. *johnsonii* MH-68, *and L*. *reuteri* GL-104 (2.5 × 10^9^ CFU capsule^-1^) could regulate blood glucose levels by activating glucose transporter 2 (GLUT2)-mediated pathway [10]. The transmembrane carrier protein GLUT2 facilitates glucose transportation across cell membranes, which is highly expressed in hepatocytes, pancreatic β-cells, and epithelial cells in the intestinal mucosa and kidney. Thus, GLUT2 may play a crucial role in plasma glucose absorption and insulin secretion [35, 36] Α decline in GLUT2 levels in pancreatic β-cells is usually observed in diabetic animal models and human patients [37]. Our results also showed that Probioglu^TM^ reduced the levels of TG (mg dl-1), TC (mg dl-1), LDL (mg dl-1) and Urea (mg dl-1) (Table 3).

Probioglu^TM^ also attenuated STZ-induced β-cell death and increased β-cell mass (Fig 3). It is known that proinflammatory cytokines can cause pancreatic β-cell failure and destruction in diabetic patients [38]. Our probiotic formula may prevent apoptotic destruction of β-cells by diminishing the proinflammatory cytokines TNF-α, IL-1β, and IL-6 (Table 4).

Previous studies have shown that oxidative stress may lead to the disruption of normal β-cell function: oxidative stress activates the c-J N-terminal kinases pathway to promote β-cell in diabetes [39]. The improved levels of serum anti-oxidative SOD, MDA, and GSH after Probioglu^TM^ administration may lead to β-cell protection.

Ricardo Beltramede Oliveira et al. revealed that High Fat Diet (HFD) intake would impaired tight junction protein structure at early stage of T2DM [40]. Thus, we tested tight junction protein expression in mRNA level by treating Probioglu^TM^ to intestinal Caco-2 cell. The result present that Probioglu^TM^ would significantly elevate mRNA level of Occludin, JAM-A and ZO-2 by comparing to medium control [41]. S1 Fig in S1 Text revealed Probioglu^TM^ would significantly elevated tight junction protein expression in mRNA level (Occludin, JAM-A and ZO-2) in Caco-2 cell model.

Besides, researchers had demonstrated exercise could ameliorate T2DM induced intestinal SFCA concentration decline in mice model [42]. It is reported that probiotic secreting SCFA would improve glycemic control among T2DM patients [43]. Certain probiotic strains including *Lactobacillus rhamnosus* GG and *L*. *gasseri* PA 16/8, *Bifidobacterium longum* SP 07/3 and *B*. *bifidum* MF 20/5 are able to produce acetate and propionate, but can’t generate butyrate [44]. In this study, we further tested the SCFA and MCFA levels generated by Probioglu^TM^. The Probioglu^TM^ consisting of viable probiotic strains AP-32, CP-9, GL-104 and MH-68 were cultured overnight in MRS medium. Collecting supernatants of individual strain then analyzing SCFA and MCFA contents by HPLC [45]. The functional SCFA including acetic acid, propionic acid and butyric acid were detected in individual strain of AP-32, CP-9, GL-104 and MH-68, which may contribute to mediate glycemic index at present study (S1 Table in S1 Text). However, further experiments should validate how probiotic secreting SCFA regulates blood glucose level and protects beta cells in animal model in the future.

## Conclusions

Overall, we showed that injections of low-dose STZ 10–20 mg/kg with a high-energy diet could successfully induce hyperglycemia and cause damage to beta cells of the test pancreas. However, the Probioglu^TM^, containing *L*. *salivarius subsp*. *salicinius* AP-32, *L*. *johnsonii* MH-68, *and L*. *reuteri* GL-104, alleviated the symptoms of type-2 diabetes in STZ-treated rats by protecting the function of β cells and stabilize the glycemic levels. Additional type-2 diabetes animal models, such as Leprdb/db mouse, NZO mice, and several non-obese animal models, may be needed to validate the benefits of Probioglu^TM^. In addition, the molecular mechanism of β cell protection and clinical study by Probioglu^TM^ needs further investigation.

## Supporting information

S1 Text (DOCX)
