# Supplementary material for: Multi-strain probiotic supplement attenuates streptozotocin-induced type-2 diabetes by reducing inflammation and β-cell death in rats
Source: PLoS One. 2021 Jun 24;16(6):e0251646. doi: 10.1371/journal.pone.0251646 (PMC8224959; doi:10.1371/journal.pone.0251646)
Supplement: S1 Text — (DOCX) [file pone.0251646.s001.docx]

**S1 Text. Supplementary figures and tables**

**S1 Fig. Tight junction protein expression in mRNA level.**

Seeding culturing 3×10^5^ Caco-2 cells (ATCC, Manassas, Virginia, USA) on a 6-well cell culture dish (Corning®, Merck, Darmstadt, Germany). Culturing cells with DMEM medium (Thermo, Waltham, Massachusetts, USA) for 11 days. Washing out DMEN medium with PBS (AAT Bioquest, Sunnyvale, CA, USA) twice, then adding 9×10^8^ CFU probiotic bacteria (Probioglu^TM^) in 3 ml DMEM medium without antibiotics to cell culture dish for 6 hours. Washing out DMEN medium with PBS twice then adding 500 μl Trizol (Invitrogen™, Waltham, Massachusetts, USA) to each well for extracting mRNA. Inverting extracted mRNA into cDNA with High-Capacity RNA-to-cDNA™ Kit (Thermo, Waltham, Massachusetts, USA). Performing real-time PCR (QuantStudio 6 Pro Real-Time PCR Systems, Thermo, Waltham, Massachusetts, USA) with the PowerTrack™ SYBR Green Master Mix (Thermo, Waltham, Massachusetts, USA) and specific primer of targeted gene. The specific primer sequences were listed as follow: Forward Primer sequence for ZO-1 was CGAGTTGCAATGGTTAACGGA；Reverse Primer sequence for ZO-1 was

TCAGGATCAGGACGACTTACTGG；Forward Primer sequence for ZO-2

GCCAAAACCCAGAACAAAGA；Reverse Primer sequence for ZO-2 was ACTGCTCTCTCCCACCTCCT；Forward Primer sequence for Claudin was

TTCGTACCTGGCATTGACTGG；Reverse Primer sequence for Claudin was

TTCGTACCTGGCATTGACTGG；Forward Primer sequence for Occludin was

TCAGGGAATATCCACCTATCACTTCAG；Reverse Primer sequence for Occludin

Was CATCAGCAGCAGCCATGTACTCTTCAC；Forward Primer sequence for

JAM-A was AGCCAGATCACAGCTCCCTA；Reverse Primer sequence for

JAM-A was TGGATGGAGGTACAAGCACA；Forward Primer sequence for

Actin was AGAGCTACGAGCTGCCTGAC；Reverse Primer sequence for

Actin was AGCACTGTGTTGGCGTACAG；Forward Primer sequence for GAPDH was GAAGATGGTGATGGGATTTC；Reverse Primer sequence for GAPDH was

GAAGGTGAAGGTCGGAGT. Statistical analysis was performed using GraphPad Prism software (GraphPad, USA). The two-tailed t-tests were used to analyze differences between groups. Results with p values of less than 0.05 were considered significant [1].

**S1 Table: The Probioglu^TM^ consisting of viable probiotic strains AP-32, CP-9, GL-104 and MH-68 generates short-chain fatty acids (SCFA) in vitro.**

|  | |  | **AP-32** | **CP-9** | **GL-104** | **MH-68** |
| --- | --- | --- | --- | --- | --- | --- |
| **Short-Chain**  **Fatty Acids (SCFA)** | Acetic acid (µM) | | 55758.1 | 129452 | 80091.4 | 67697.6 |
|  | Propionic acid (µM) | | 184.8 | 551.9 | 237.2 | 203.6 |
|  | Butyric acid (µM) | | 85.3 | 101.1 | 93 | 84.8 |
|  | Isobutyric acid (µM) | | 78 | 179.8 | 77.5 | 95.5 |
|  | Valeric acid (µM) | | 14.7 | 10.2 | 13.4 | 10.8 |
|  | Isovaleric acid (µM) | | 26.7 | 31.2 | 28.4 | 27.6 |
| **Medium-Chain Fatty Acids (MCFA)** | Hexanoic acid (µM) | | 19.6 | 15 | 19.5 | 15.9 |
|  | Heptanoic acid (µM) | | 7.3 | 2.5 | 4.9 | 3.9 |
|  | Octanoic acid (µM) | | 21.2 | 13.8 | 20.2 | 11.4 |
|  | Decanoic acid (µM) | | 3039 | 2710.5 | 7173.3 | 9251.6 |

In this study, we further tested the SCFA and MCFA levels generated by Probioglu^TM^. The Probioglu^TM^ consisting of viable probiotic strains AP-32, CP-9, GL-104 and MH-68 were cultured overnight in MRS medium. Collecting supernatants of individual strain then analyzing SCFA and MCFA contents by HPLC.

| **S2 Table: Discrepancies in blood glucose levels with varying doses of STZ (Goyal et. al, 2016)** | | | |
| --- | --- | --- | --- |
| **Dose of STZ and rote of administration** | Animal | Efficiency (blood glucose level) | Comments |
| **30 mg/kg**  **(twice/day, i.p.)** | Wistar rats | 250 mg/dL | Stable hyperglycemia |
| **HFD + 35 mg/kg (single i.p./i.v.)** | Sand rat, C57/BL6J mouse, Spiny mouse | 250-350 mg/dL | No change in body weight, stable hyperglycemia |
| **45 mg/kg**  **(single i.p./ i.v.)** | Wistar rats | 300-400 mg/dL | Cardiovascular complications, Decrease in body weight |
| **55 mg/kg**  **(single i.p./ i.v.)** | Albino rats | 450 mg/dL | decrease body weight, nephrotoxicity |
| **65 mg/kg**  **(single i.p./ i.v.)** | Albino rats | 350-500 mg/dL | Gastric ulcerations, decrease in muscle mass and bone volume, reproductive dysfunction, nephrotoxicity, bronchial exacerbations |
| **>70 mg/kg**  **(single i.p./i.v.)** | Wistar rats | >500 mg/dL | Lethal end point |

**References:**

1. Guo, W., Wang, P., Liu, Z. H., & Ye, P. Analysis of differential expression of tight junction proteins in cultured oral epithelial cells altered by Porphyromonas gingivalis, Porphyromonas gingivalis lipopolysaccharide, and extracellular adenosine triphosphate. International journal of oral science, 2018, 10(1), e8-e8.
